# Research Trends in Wireless Visual Sensor Networks When Exploiting Prioritization

**DOI:** 10.3390/s150101760

**Published:** 2015-01-15

**Authors:** Daniel G. Costa, Luiz Affonso Guedes, Francisco Vasques, Paulo Portugal

**Affiliations:** 1 DTEC-UEFS, State University of Feira de Santana, Feira de Santana 44036-900, Brazil; 2 DCA-CT-UFRN, Federal University of Rio Grande do Norte, Natal 59072-970, Brazil; E-Mail: affonso@dca.ufrn.br; 3 IDMEC-FEUP-UP, University of Porto, Porto 4200-465, Portugal; E-Mail: vasques@fe.up.pt; 4 INESC TEC-FEUP-UP, University of Porto, Porto 4200-465, Portugal; E-Mail: pportugal@fe.up.pt

**Keywords:** wireless visual sensor networks, global-level prioritization, sensing relevance, network optimization

## Abstract

The development of wireless sensor networks for control and monitoring functions has created a vibrant investigation scenario, where many critical topics, such as communication efficiency and energy consumption, have been investigated in the past few years. However, when sensors are endowed with low-power cameras for visual monitoring, a new scope of challenges is raised, demanding new research efforts. In this context, the resource-constrained nature of sensor nodes has demanded the use of prioritization approaches as a practical mechanism to lower the transmission burden of visual data over wireless sensor networks. Many works in recent years have considered local-level prioritization parameters to enhance the overall performance of those networks, but global-level policies can potentially achieve better results in terms of visual monitoring efficiency. In this paper, we make a broad review of some recent works on priority-based optimizations in wireless visual sensor networks. Moreover, we envisage some research trends when exploiting prioritization, potentially fostering the development of promising optimizations for wireless sensor networks composed of visual sensors.

## 1. Introduction

Wireless sensor networks (WSN) have been employed as an effective resource for a series of monitoring applications, providing information, such as humidity, pressure, temperature and luminosity, among many others [1,2]. For those networks, when sensor nodes are equipped with low-power cameras, visual information can be retrieved from the monitoring field, potentially enriching scalar sensed data [3,4]. In short, wireless visual sensor networks (WVSN) consist of a number of resource-constrained battery-operated nodes, where some or all of them are endowed with a low-power camera [3,4].

The nature of data transmission in wireless visual sensor networks has fostered the development of priority-based approaches for high-performance monitoring. In general, wireless sensor networks are constrained in processing, memory and energy resources, turning visual data gathering, handling and transmission into a challenging task. As transmission of still images and video streams may be too stringent for resource-constrained sensor networks, some prioritization approaches may be employed to soften the transmission requirements for lower relevant data. Basically, as lower relevant data is less significant for the overall monitoring quality, they may be sometimes discarded to save resources. In this context, a common approach is the exploitation of the characteristics of the employed coding technique, which is significant only for the considered transmission flow. Some image and video codecs produce data with different relevancies for the reconstruction process, opening feasible possibilities for optimizations. Many recent works have proposed promising solutions exploiting such local-level prioritization parameters for higher efficiency.

On the other hand, the nature of visual monitoring has some additional characteristics that can be exploited to achieve a new perception of prioritization in WVSN. Visual sensors collect information following a directional sensing model, according to their field of view (FoV). Actually, the FoV is a sector-like visible region emanating from the camera [5,6], defining a direction of viewing (the camera's pose). This characteristic allows that even close sensors may view different targets or areas, turning the effective monitoring behavior of each visual source node into a function of the sensors' poses and monitoring requirements, instead of only the network configurations after deployment. As a result, the quality of visual sensor networks will be also a function of how well an area of interest or a set of targets is viewed by source nodes, and such quality depends on the actual application requirements. In fact, this particularity can be used to define novel global prioritization parameters, where prioritization is performed considering what are transmitting sources instead of the inner characteristics of the transmission flows resulting from a particular coding algorithm.

The work in [7] formulated the concept of sensing relevance as priority indexes that indicate the importance of each visual source node for the whole monitoring function of an application. The sensing relevance concept defines a global-level prioritization approach that is significant for the entire network, requiring centralized computing at the sink side [7]. The final computed sensing relevance can then be exploited for different optimization mechanisms, achieving higher performance when data transmitted from high-relevant source nodes are prioritized over data from lower relevant sources. In a superficial analysis, it exploits the same principle of prioritization based on multimedia coding, but the optimization algorithms will consider the transmission sources and not the transmitted data.

Prioritization of source nodes by their sensing relevancies may happen in different ways. As an example, visual sensors that view critical areas may be assigned to higher priority levels, since they will retrieve more significant data for the monitoring functions of the applications. However, different approaches may also be taken. In [8], a global-level prioritization approach is proposed where the priorities of visual source nodes are established based on the occurrence of critical events, such as a bomb explosion. In such a case, source nodes viewing the affected area are more critical for the monitoring application, and thus, their packets should be prioritized.

We then discuss in this paper some promising investigation areas where local and global-level prioritization parameters could be exploited for higher efficiency in WVSN. In other words, we envisage potential uses of prioritization parameters that were not considered before, indicating promising research directions. Additionally, we will introduce key optimization issues when exploiting such parameters. Doing so, we expect to guide new investigation efforts in this area.

The remainder of this paper is organized as follows. Section 2 states the fundamentals of prioritization in wireless visual sensor networks. Then, Section 3 surveys recent works exploiting local-level prioritization parameters. A survey of global-level prioritization is presented in Section 4. Section 5 discusses relevant research trends for optimizations in wireless visual sensor networks when exploiting local and global-level prioritization. Some key optimization issues are presented in Section 6, followed by conclusions and references.

## 2. Fundaments of Prioritization in WVSN

The stringent requirements of visual data transmissions have demanded innovative optimizations for wireless sensor networks in order to achieve higher efficiency. For such optimizations, prioritization is an effective way to enhance performance when some elements of the networks may be differentiated by their significance. Optimizations centered on prioritization will improve efficiency when the network behaves in a different way according to the considered priority paradigm. Additionally, although some quality loss may be perceived when low relevant data is not prioritized, generally applications will be less sensitive to such data when a reasonable prioritization framework is defined.

When designing priority-based optimizations, two different aspects must be considered. The first aspect is the prioritization parameter. The second aspect is how such a parameter will be exploited during the network operation. For wireless visual sensor networks, different approaches for both aspects may be defined.

Many prioritization parameters may be defined in wireless visual sensor networks. Transmitted data may be differentiated by their type (scalar, audio, image or video), coding algorithm, real-time requirements, criticality, confidentiality, among others. On the other hand, source nodes may be differentiated by their hardware capabilities, current energy resources, sensing relevance, routing relevance and many other characteristics.

In general, the chosen prioritization parameter may have a local or global scope. A local scope is related to a particular transmission flow, from a particular source node. In a different way, a global scope relates to a significance context that is valid for the entire network. As an example, a multimedia source node may transmit data packets where all image packets are more relevant than the remaining packets within this scope. However, all image packets from all source nodes may also be more relevant than all other packets or, for example, only image packets from source nodes with lower energy resources.

Table 1 presents some common prioritization parameters for wireless visual sensor networks.

The second aspect is how the prioritization parameter will be exploited to enhance the network performance. Generally, a priority-based optimization is aimed at some quality of service (QoS) or quality of experience (QoE) goal. In a rough definition, the quality of service is an indication of the expected quality of communications, which may be associated with characteristics, such as throughput, latency, jitter and packet error rates. Thus, a typical priority-based optimization could adapt error recovery, routing, congestion control, security and the energy consumption pattern in the wireless sensor network operation, just to cite a few approaches.

Besides QoS, wireless visual sensor networks may be concerned with the QoE of the performed monitoring functions, which is a subjective metric. In short, the quality of experience indicates the perception of the sensed data for the application and its users. Monitoring applications may define minimum acceptable levels for the QoE, which will be related to the quality of the retrieved data according to the overall monitoring requirements. In other words, the expected quality of the retrieved data is a function of the applications and not the networks. For a priority-based optimization, the network could be adapted to preserve more significant data for the monitoring functions of the applications, when data packets are transmitted over error-prone wireless links. Additionally, as only high relevant data is preserved, energy consumption and the processing burden are reduced with respect to the application QoE.

Optimizations centered on QoS or QoE may be concerned with a specific transmission flow or the entire network, according to the considered prioritization parameters. When they are related to a single transmission flow, which we define as a local-level scope, the employed optimization mechanisms are valid only for that context and do not affect other transmissions. On the other hand, we define the context of global-level prioritization when the considered parameters are valid for the entire network. In the first case, we may have, for example, packets with different priorities inside the same transmission flow. For the second case, packets from different source nodes may have different priority levels among them.

There are then some key design issues that must be considered when defining priority-based optimizations, expressed as follows:

(1) Monitoring applications in WVSN may select prioritization parameters that will guide the network operation. In general, such prioritization may be based on a local or a global perspective of the sensor network. For the local-level scope, some characteristics of the employed data coding algorithm may be considered or even the different data types transmitted from the same sensor endowed with multiple sensing units. On the other hand, the prioritization may be based on a global-level scope, with each sensor having a relevance that is meaningful for the entire network.
(2) For each chosen parameter, a set of possible values must be defined, which may be a range of numeric indexes or just a subjective classification (e.g., low, medium and high). Those are the priority levels that will guide network optimizations. Obviously, prioritization is only valid if different priorities may be defined for different source nodes and/or data packets. If every considered datum has the same relevance, priority-based optimizations cannot be accomplished.
(3) After defining the prioritization approach, optimization mechanisms may be created to exploit the priority levels, for example adapting transmission, error recovery, congestion control, data coding or routing procedures. Selecting the most appropriate optimizations is not straightforward and the particularities of each sensor network and monitoring applications should be considered.

This basic methodology can be very beneficial for wireless visual sensor networks, and there are some works that have proposed feasible solutions in this area. However, there are still few works that have investigated global-level prioritization, leaving open still many research issues.

## 3. Local-Level Prioritization in WVSN

The resource-constrained nature of wireless sensors, which are designed to be cheap and autonomous to allow massive deployment, is opposed by the stringent requirements of visual data transmission, turning efficiency into a major concern for WVSN. Transmission of image snapshots and video streams can rapidly deplete the energy resources of the nodes. Moreover, the network may face congestion and significant packet losses when transmission flows cannot be satisfactorily handled. This complex scenario has required different levels of network optimizations, and many works in recent years have addressed performance enhancement for these networks.

A common approach to optimize WVSN is to exploit some characteristics of an active transmission flow [9]. As depicted in Table 1, such optimizations usually are based on the type of sensed data (audio, image, video or scalar data) or the nature of the multimedia coding technique. There are some recent works in the literature that have proposed practical solutions based on these prioritization strategies.

The work in [10] finds the best paths (lower latency) for multimedia streaming in wireless sensor networks, considering a set of available node-disjoint paths. The original media stream is split into image and audio, giving to each resulting sub-stream a particular priority. The best paths are then used by the higher priority sub-stream, allocating the remaining paths to the lower priority sub-stream. In [11], an adaptive relaying approach is proposed, where data packets are relayed by intermediate nodes according to their local-level relevance and the energy resources of the nodes. In that work, if they are running low in energy, only high-relevant packets are relayed to the next node toward the sink.

In a different way, characteristics of the multimedia data coding can be used to define a local-level prioritization approach. In [12], the authors propose a full-reliable image transmission mechanism for high-priority discrete wavelet transform (DWT) sub-bands, while the remaining DWT sub-bands are transmitted in a semi-reliable mode. The relevancies of the DWT sub-bands are assigned according to the way images are reconstructed at the sink side. Exploiting this relevance concept, intermediate nodes in [12] may silently drop low relevant packets according to their current energy level, potentially saving energy and prolonging the network lifetime. The work in [13] exploits DWT coding in a different way, where the coding relevancies are considered when the network faces congestion. The idea is to reduce the amount of information that flows through the network, but such a reduction is concentrated in lower relevant DWT sub-bands. Reduction of the overall amount of data to transmit exploiting DWT coding is also performed in [14]. The work in [15] exploits a different image coding technique, but defines an equivalent strategy for data prioritization. In [15], the unequal importance between image-pixel-position information (p-data) and image-pixel-value information (v-data) is exploited for prioritization. As the p-data is more relevant, it receives more protection by an unequal error protection (UEP) mechanism, potentially achieving energy savings when fewer data are transmitted.

Video streaming may also be exploited when defining local-level priorities. The work in [16] considers visual sensors that are transmitting video frames encoded by H.264, a video codec that employs previously encoded frames as a reference for motion-compensated prediction. As there are some frames that are more relevant for the reconstruction of the original video than other frames, the authors propose a mechanism to reduce the transmission rate, discarding lower relevant packets. A similar investigation is conducted in [17], where the characteristics of the MPEG-2 codec are exploited to assure reliable transmissions for only the most relevant data.

In fact, many optimization mechanisms have addressed different aspects of the communications in wireless visual sensor networks [9,18]. Whatever the case, the central idea is to achieve higher efficiency with the lowest loss on the monitoring quality. Typically, there will be a tradeoff between the network performance and the monitoring quality, but we can expect that most applications can tolerate some quality loss if the overall network performance is enhanced. In such a way, the differentiation of parts of the visual data according to the employed coding technique is a reasonable way to minimize that quality loss.

## 4. Global-Level Prioritization in WVSN

Visual data prioritization is indeed relevant, but equally penalizes all source nodes. However, source nodes may have different relevancies for the applications, and we may want to assure high quality of the transmitted data from the most relevant sources. Thus, a prioritization strategy that is centered on the source nodes, instead of the transmitted data, may be desired.

In general, global-level prioritization in WVSN is focused on the way visual sensors gather information from the monitored field. Actually, visual sensors gather information following a directional sensing model, where the concept of vicinity is valid only for communications. The area sensed by camera-enabled sensors is defined as the field of view [5,6], a sector-like area that is defined by a viewing angle, a sensing range and an orientation [19]. When performing a 2D modeling, the FoV is commonly approximated to an isosceles triangle for simplicity, but 3D modeling is also possible [5,6].

Since the visually covered area depends on the camera's FoV instead of only the sensors' positions, the significance of the visual source nodes is a characteristic of the sensors themselves, which, in turn, depends on the application monitoring requirements. In this context, such differentiation may be exploited as a prioritization parameter, according to the potential that visual source nodes have to provide relevant information for the monitoring functions of the applications. Since global-level prioritization is not associated with the network topology and sensors positioning, the same exact sensor network may have source nodes with different priorities, depending on the considered application requirements.

There are still few works that have proposed the use of global prioritization in wireless visual sensor networks to enhance the overall monitoring performance. Additionally, fewer are the methodologies to define global-level prioritization parameters. In [7], the concept of sensing relevance as a practical mechanism to provide global-level relevance indexes to visual source nodes is proposed. The visual sources are classified into five different groups of relevance: Irrelevant, low relevance, medium relevance, high relevance and maximum relevance groups. Based on the assigned group of relevance, each visual source node computes a sensing relevance (SR) index considering complementary information, such as residual energy, enabled coding techniques and available viewing capabilities (zooming, resolution, mobile capability, *etc.*). Table 2 details the groups of relevance and the associated range of SR, as described in [7].

The assignment of visual source nodes to groups of relevance and the consequent computation of the SR are described in [7]. In short, the assignment of source nodes to groups of relevance is expected to be performed in a centralized unit with a global view of the network, usually at the sink side. Such centralized processing may consider user perceptions of the retrieved information, when visual data quality is defined by QoE parameters [20,21]. Another approach is the computing of visual patterns to identify relevant information. At last, it is defined that groups of relevance may be processed according to the monitoring of regions of interest. Applications may define virtual regions where relevant data are more likely to be retrieved, and sensors that can view those regions will be assigned to a particular relevance level, for example considering a minimal percentage of FoV over the defined regions.

**Figure 1.**
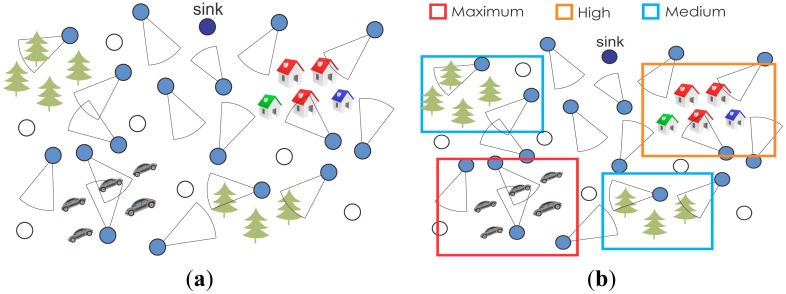
Exploiting global-level prioritization in wireless visual sensor networks. The monitoring of cars is more relevant in this example, but other configurations are possible. WVSN, wireless visual sensor networks. (**a**) A typical WVSN; (**b**) A WVSN exploiting global-level prioritization.

Figure 1 presents a typical wireless visual sensor network where regions of interest are defined according to the monitoring requirements of a particular application. Regions of interest must not intersect, and areas that are not covered by any region are assumed to have low relevance for the application. All sensors that belong to the same group of relevance and, thus, can view regions of interest with the same relevance are equivalent for the monitoring application, but they may have different global prioritization parameters according to characteristics of each visual sensor.

The sensing relevance of the source nodes will be deeply related to the overall monitoring quality. If more information from higher relevant source nodes is preserved, the monitoring quality is not likely to be significantly degraded. On the other hand, applications can typically tolerate some loss of low-relevant information. Such a prioritization approach may not be related to real-time or reliability requirements or even the criticality of transmitted data, since the ultimate prioritization is centered on the data relevance for the application.

The framework proposed in [7] considers the data relevance as an expectation, since the application expects that high-relevant source nodes will provide high-relevant data. However, global-level prioritization may be also related to a current state of relevance. In [8], the authors propose global-level priorities to be assigned according to the occurrence of some event of interest. When a critical event is detected, all visual sensors that can view it are assigned to a global-level priority, which also considers the criticality related to areas of the monitored field. Defining a monitoring relevance (MR) index ranging from zero to two, where two is assigned to the highest relevant sources, the network can provide prioritized services to transmission flows from more relevant sources, potentially assuring that critical events will be monitored with higher visual quality and lower delay. The identification of critical events in [8] is performed by traditional scalar sensors, and differently from [7], decisions are made closer to where events happen. This is the reason why event detection is performed by scalar sensors: relevant information can be detected faster when compared with centralized processing at the sink.

In both cases, there will be source nodes with higher priority for data sensing and transmissions, considerably changing the way sensor networks deliver information to the sink. However, each source node can also define a local prioritization approach for its own transmission flow.

Recently, some works proposed optimizations in wireless visual sensor networks exploiting global-level prioritization parameters. In [22], the transmission frequency of source nodes is established according to the sensing relevancies of the active visual sources, achieving energy savings when only higher relevant sources transmit data at a high frequency. In [23], an energy-efficient retransmission mechanism to assure full reliability only to packets transmitted from high-relevant sources was proposed. The same concept of global-level priority-based optimization was exploited in [24], where high-relevant packets are forwarded through paths with lower average end-to-end delay, which are assumed to be the best paths for real-time applications. The work in [25] exploits the sensing relevance concept to enlarge the network lifetime when intermediate nodes with low energy level forward only packets with higher relevance, saving energy when low-relevant packets are silently discarded. Finally, the work in [26] proposes packet-level redundancy for reliability in WVSN, but exploiting global-level prioritization to provide differentiated reliability services according to the packets' origins. All of these works bring significant contributions to wireless visual sensor networks, exploiting global-level prioritization parameters, but many novel optimizations can still be envisaged.

Optimization approaches based on global-level parameters will be centered on the prioritization of visual source nodes, which might be mainly accomplished considering the relevancies of source nodes due to their hardware characteristics (as cameras' resolution and zooming capabilities), according to the relevance of the targets being viewed, according to area monitoring or even considering the monitoring of critical events. In fact, the sensing relevance concept proposed in [7] and the adaptive monitoring approach presented in [8] are practical frameworks that can be considered for general-purpose WVSN, and some promising optimizations have been recently proposed exploiting those prioritization strategies. However, many other prioritization frameworks might be proposed, centered on information, such as sensor deployment, security, mobility, camera calibration, among others. In fact, any parameter that has global significance and that can be used to differentiate transmitted data can be exploited in different optimization approaches. Although we are not particularly concerned with how such prioritization frameworks could be defined, we will discuss many promising optimizations that could exploit such frameworks.

## 5. Research Trends for Priority-Based Optimizations

Optimizations based on prioritization parameters may bring significant results for wireless visual sensor networks, and some recent works have proposed promising contributions when exploiting prioritization. However, many different optimization approaches can still be envisaged, further enhancing the performance of these networks.

Energy is critical in wireless visual sensor networks, and thus, most investigation works will be concerned with energy efficiency. However, some key aspects, such as real-time transmission, reliability, availability and security, are also relevant, but many optimizations focused on these aspects will present better performance when the expected goals are achieved with some energy savings. In this context, global-level prioritization is valuable, because it allows differentiated treatment of visual source nodes and their transmitted packets, but local-level prioritization can also be exploited.

In order to attain high efficiency, protocols and algorithms of the MAC, network, transport and applications layers can operate in a cooperative way that disrupts the conventional data flow [9]. Such cross-layer design is highly beneficial for wireless visual sensor networks, especially when prioritization approaches are employed. In fact, most optimization approaches proposed so far are designed under cross-layer premises, and we expect that most future optimizations will follow this same trend.

In this section, we discuss promising areas where prioritization parameters could be exploited for higher efficiency, potentially guiding investigation efforts in this area. Some of these areas are best fit for global-level prioritization, but others may be considered for both paradigms.

### 5.1. Sensor Deployment

Wireless visual sensor networks will be typically deployed over a monitored field for some kind of sensing function, according to the application monitoring requirements. In fact, sensor nodes may be randomly or deterministically deployed. In a random deployment, sensors will be scattered over a target area, which may result in regions densely or sparsely covered by visual sensor nodes. On the other hand, deterministic positioning of visual sensors allows optimal sensing coverage of the monitoring field, but it may be unfeasible in hazard or hard access areas [27].

Sensors deployment may be optimized exploiting the relevance of visual source nodes. For example, in regions of interest with higher relevance for the application, more sensors or sensors with better resources could be deployed to enhance the overall monitoring quality. Massive deployment in regions of interest with higher relevance for the current application may result in more redundant nodes on average, which could be employed to enhance the availability level of the performed visual monitoring. In this context, if sensors are being airdropped, the previous knowledge of the regions of interest could guide the nodes' deployment, although the resulting configuration cannot be predicted due to weather conditions or hardware damage when landing. Moreover, such previous knowledge could be also considered when performing deterministic deployment.

The work in [28] proposes directional *K*-coverage, which estimates the probability that all targets in the monitored field are viewed by *K* sensors. This metric is concerned with the probability of achieving a directional *K*-coverage configuration after a random deployment, and it is shown in [28] that higher values for directional *K*-coverage are achieved when more sensors are deployed. Thus, we could expect that denser deployment in more relevant regions of interest would result in better coverage of those regions, potentially benefiting visual monitoring applications centered on prioritization based on sensing redundancy. In such a way, a deployment approach exploiting global-level prioritization would bring significant contributions to WVSN optimization mechanisms, but further investigation in this area is still required.

### 5.2. Load Balancing

Sometimes, active source nodes may be connected to the sink of the network through more than one transmission path. A natural thought that arises is that source nodes may employ all paths to increase the available transmission bandwidth or to enhance the fault tolerance when some path fails (due to energy depletion, node malfunctioning, high congestion or excessive packet dropping). A load balancing strategy would split the transmission flow through all paths in order to increase the available bandwidth, but it is a complex task. Generally, load balancing may increase the energy consumption of the network, requiring proper planning and management, but it may be required for transmissions of high-resolution visual data.

Source nodes may be connected to the sink through braided paths, where at least one intermediate node belongs to more than one transmission path. In those cases, such intermediate nodes may receive too much traffic, which may considerably increase their energy consumption and congestion. In this context, we could exploit local-level prioritization, assuring that only the most relevant data packets from a particular source node would be relayed to the next hop or forwarded to the best transmission paths in terms of some QoS parameter (latency, error rate, throughput, *etc.*). In a different perspective, only data packets from the most relevant source nodes could be prioritized, when global-level prioritization is considered.

Table 3 proposes a reasonable optimization approach for load balancing based on global-level prioritization, considering five generic levels of relevance. The relevance of the sources will indicate how many paths the corresponding visual source node may consider for transmissions, in the case that there are many available paths. If the required transmission bandwidth is greater than the sum of the available bandwidth of the selected paths, the source node must discard (low-relevant) packets until the transmission flow can fit into the available bandwidth.

Besides braided paths, source nodes may be connected to the sink through disjoint paths. Those paths do not share any intermediate nodes, and thus, they can be thought of as an exclusive resource for source nodes. Sometimes, disjoint paths may be composed of some visual sensors, which may be transmitting or only acting as relay nodes. In such a way, more relevant visual sensors that compose *ad hoc* paths should receive less traffic for relaying, potentially enlarging their lifetime when less energy is consumed. The work in [29] proposed a coverage-aware load balancing strategy aimed at the reduction of the traffic over paths composed of more visual sensors. Although promising, that work could be extended to consider the sensing relevance of each visual sensor. The following equation presents the algorithm to compute a fair load balancing as proposed in [29], where *L*_(_*_p_*_)_ is the load in path *p*, *B* is the required source bandwidth, *B_1_* is the bandwidth allocated to paths composed of only scalar sensors and *PC*_(_*_p_*_)_ is the sum of the coverage index of the nodes that compose the path *p*, for a network composed of *P* node-disjoint paths. In that work, the coverage-index is one when the node is equipped with a low-power camera and zero when it is a scalar sensor.

(1) $$L_{\left(p\right)}=\frac{{\left(B-B_{1}\right)}^{2}}{PC_{\left(p\right)}\times \sum_{i=1}^{P}\left(\frac{B-B_{1}}{PC_{\left(i\right)}}\right)}$$

*PC*_(_*_p_*_)_ could be replaced by *PSR*_(_*_p_*_)_, which would be the sum of global-level priorities of all visual sensors that compose the path *p*. Doing so, the visual sensors would be preserved according to their global relevance, and the number of visual sensors that composed the path would not be relevant, but their priorities would.

### 5.3. Error Recovery

Sensor nodes are usually interconnected by error-prone links, where bit-errors are common, potentially degrading the application monitoring quality. Hence, when transmitting visual data packets over error-prone wireless links, packets can be corrupted. In wireless visual sensor networks, the unique view of camera-enabled sensors over the monitored field [6] turns the recovery of lost packets into a required service. Additionally, some parts of the visual information transmitted from source nodes may be extremely relevant for the decoding process at the sink side, for example when coding techniques, such as JPEG2000 and H.264, are employed.

Generally, packet corruption can be recovered employing retransmission or redundancy techniques [26,30]. Retransmission of corrupted packets assures that a new copy of the lost packet will be retransmitted in an end-to-end or hop-by-hop fashion. On the other hand, redundancy will add information in advance, either into data packets (as an additional header) or creating replicated packets. In a general way, local and global-level prioritization parameters could be exploited to optimize the way corrupted packets will be recovered.

In [23], a semi-reliable transmission mechanism where only high-relevant packets are retransmitted if corrupted, assuring average lower energy consumption over the network, was proposed. That work proposed hop-by-hop retransmissions as an effective approach for wireless visual sensor network, but it was assumed that every single packet needed to be acknowledged by the next hop toward the sink. However, it could still be extended considering a priority-based retransmission mechanism, where acknowledgments for blocks of messages would be employed for higher efficiency.

A similar idea arises for the work described in [26], which exploits the global-level prioritization concept of [7] when defining the number of copies to transmit for each original data packet. Higher relevant source nodes transmit more replicated packets, increasing the average quality for the most relevant data while saving energy over the network. That work could be extended considering the replication of only parts of the transmitted data, for example assuming a DWT coding.

Besides retransmissions and the transmission of replicated packets, error recovery can be performed by forward error correction (FEC). It is performed adding redundant information into data packets with different levels of complexity, where corrupted packets may be recovered by processing the codes [31]. Thus, a reasonable global-level prioritization approach would employ different configurations of correction codes according to the priorities of source nodes. A popular correction code is the Reed-Solomon [31], which can detect any combination of up to *t* erroneous symbols when *t* redundant information is added. The code rate indicates the ratio between the number of original bits and the total number of bits to transmit (including redundancy), and high code rates mean that lower redundancy was added (and thus, *t* is lower). When high error resilience is desired, the code rate must be low, requiring the addition of more redundant information. However, it may also imply higher energy consumption over the transmission paths.

One could exploit global-level prioritization assuring that only higher relevant source nodes would transmit packets with more redundant information. Table 4 presents a practical exploitation of this concept for error recovery, assuming five generic prioritization levels.

**Figure 2.**
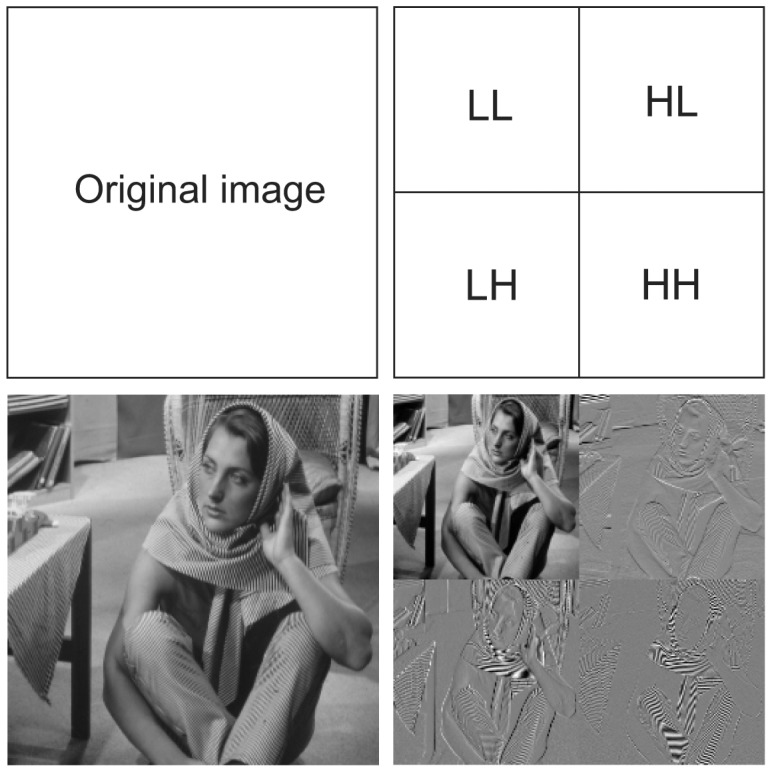
A one-level 2D DWT.

Local-level prioritization parameters, such as provided when employing DWT coding, can be exploited together with global-level prioritization in order to achieve more complex and robust optimization solutions. DWT is a wavelet transform that provides data decomposition into multiple levels of resolution. A one-level 2D DWT processes original images considering rows and columns, generating four sub-bands according to the considered filters (Low and High): LL, LH, HL and HH. The LL sub-band represents a half-sized version of the original image, but the remaining sub-bands are required to perfectly reconstruct the original image. In fact, the LL sub-band is the most relevant for the reconstruction of the original data. Figure 2 presents a schema for a one-level 2D DWT over an original image.

A more complete optimization approach could then be proposed, as expressed in Table 5, employing local and global-level prioritization, assuming as the reference a discrete global-level prioritization scheme ranging from zero to seven and a local-level prioritization based on DWT coding.

In fact, many different mappings between local and global-level prioritization parameters may be proposed, with different results in terms of coding complexity, energy efficiency and error resilience.

### 5.4. Congestion Control

Due to the nature of visual data transmissions in wireless sensor networks, intermediate nodes may become congested, resulting in buffer overflows. Communication links may also become congested when the transmission demand is too high. When event-based monitoring is performed by WVSN, events may trigger bursts of packet transmissions, turning congestion control into a challenging task.

In short, congestion may be mitigated by two different approaches: reduction of the source transmission rate or packet dropping. Reduction of the source rate is an adaptive approach that allows minimal packet losses, as proposed by some works [9,13], but it adds some complexity to the communications. When exploiting global-level prioritization parameters, a congested node could notify the visual source nodes requesting immediate reduction on the transmission rate, but those sources could behave differently according to their priorities. Table 6 presents a reasonable approach for congestion mitigation, where DWT coding is also considered as a local-level relevance parameter. We assume three generic values for global-level prioritization.

A congestion mitigation mechanism can also change the way visual data coding is processed, for example altering the number of frames per second (fps) for video codecs. Nevertheless, such changes will typically be reflected in the source transmission rate, but one could imagine a large set of mappings between global-level priorities and the source transmission behavior.

Another approach to face congestion is selective packet discarding in congested nodes. The priorities of the source nodes may be exploited to provide higher priority to packets carrying the most important information for the application, and those priorities will be considered when congested nodes need to select packets for dropping. Such an optimization was initially proposed in [7], defining that when a new packet reaches an intermediate node with a full receiving queue, a lower relevant packet should be replaced by the incoming packet. This principle may also apply when intermediate nodes are not congested, providing a mechanism for optimal packet routing. In such a way, high-relevant packets may be transmitted with lower average end-to-end delay. Another possibility is the definition of exclusive receiving queues for each of the groups of relevance proposed in [7], where the processing schedules would prioritize high relevant packets.

In general, we envisage innovative uses of global-level prioritization parameters when dealing with packet processing in intermediate nodes, potentially considering local-level priorities as a complement for higher efficiency.

### 5.5. Communication Protocols

Visual monitoring applications may have different transmission requirements, but large bandwidth, reliability, availability and timeliness are usually required. In this context, global-level prioritization can be exploited in different layers of the communication protocol stack for enhanced performance.

Clustering and packet forwarding in wireless sensor networks are highly relevant topics, and many works have proposed significant solutions in the last few years. However, many priority-based approaches can still be proposed, potentially changing the way packets are forwarded throughout the network. For example, a cluster head could prioritize source nodes with higher priority. In-network processing can also benefit from the use of local and global-level prioritization parameters. In fact, when higher relevant source nodes are prioritized, one can achieve acceptable monitoring quality with optimal energy consumption.

The time constraints of the applications depend on the expected monitoring functions, but real-time transmissions may be required by many applications. Therefore, visual source nodes with higher relevance for the applications should always have higher priority when transmitting packets. If real-time transmission is required, high-priority packets may be transmitted through paths with lower end-to-end delay in order to comply with the application time constraints. In [24], a global-level priority-based transmission approach, where high-relevant packets are forwarded through paths with lower end-to-end delay, according to the number of intermediate hops that compose the paths, was proposed. In fact, lower end-to-end delay may be expected from shorter paths [10]. Such an assumption could guide the network configurations and deployment, where the transmission paths are created according to the relevance of source nodes.

It is unlikely that wireless visual sensor networks can provide real-time transmissions for all active visual source nodes due to the constrained nature of the sensors. Thus, we can exploit global-level prioritization parameters to provide differentiated services for the source nodes. The work in [32] presents a promising cross-layer approach for real-time delivery in wireless multimedia sensor networks, where different protocols are optimized. Such approaches could be further extended when exploiting global-level priorities, since the data traffic would have different relevancies.

Communication in the MAC layer is crucial for wireless sensor networks. MAC protocols perform medium access control, which incurs energy consumption and end-to-end latency. In this case, the same principle discussed before is valid here, which is the prioritization of sources nodes for the current functions of the monitoring applications. For example, a potential optimization is a global-level priority-based schedule of packet transmissions. In fact, energy consumption in sensors depends mostly on the time the wireless radio is turned on. If the sensor is active, but has nothing to transmit or receive, energy is wasted. This idle listening situation can be minimized employing duty cycle MAC protocols, such as the T-MAC [33], which alternates the sensor nodes between active and sleeping periods for energy saving. The sleeping time in T-MAC is dynamic, but more relevant source nodes could have shorter periods of inactivation when they have data to transmit, while the lower relevant sources would have higher sleeping time. Doing so, we could assure transmissions with lower end-to-end delay for the most relevant sources, especially benefiting event-triggered monitoring applications. Nevertheless, as lower relevant nodes with higher sleeping times may also participate in multihop transmission paths, such a prioritization approach should also consider the transmission and sensing context for each node.

If sensor nodes are communicating using a MAC protocol with a “back-off time” after collisions, like carrier sense multiple access with collision detection (CSMA/CD), global-level prioritization parameters could also be exploited to assure lower back-off for higher relevant visual source nodes. Doing so, those more relevant sources would acquire the channel more often, potentially achieving higher efficiency. However, many others cross-layer optimizations can be envisaged to adapt the MAC layer [34].

Another possibility is optimization when selecting the transmission frequency and the transmission power. If sensors are deterministically deployed, the transmission power can be adjusted to optimize energy consumption, for example considering the geography of the monitored field [35]. In this case, global-level prioritization could be exploited to guide the deployment of sensor nodes and configurations in the physical layer of the protocols stacks.

### 5.6. Security

Wireless sensor networks have to deal with many security issues, where confidentiality, integrity, authentication and availability are major goals do achieve [36]. When camera-enabled sensors are deployed, the nature of visual monitoring will add new challenges for security [37], but will also bring many opportunities for optimizations based on novel prioritizations approaches.

Global-level prioritization parameters can be exploited to enhance the adopted security mechanisms in WVSN. Additionally, such exploitation can be performed differentially according to the desired goal. When addressing confidentially and data integrity, the global relevance, for example, may indicate the level of cryptography that each source node will apply over its transmission flow, where more relevant sources will employ more robust cryptography algorithms, while low-relevant sources may transmit clear data. The size of the cryptograph key may also be associated with the priority of the visual source nodes, reducing complexity when only higher relevant sources employ strong cryptography. Table 7 presents a valuable mapping between a generic global-level prioritization approach and the expected cryptography of sensed information.

Admission control can also be optimized. Each region of interest may have a proper admission policy for new sensors, where more relevant or critical areas will have a more robust admission control. In Figure 1, for example, the region of interest associated with the maximum relevance group (cars monitoring) may require a strong authentication mechanism to properly control the admission of new visual sensors, since the retrieved information is highly relevant for the overall monitoring quality. In such a case, we could restrict the admission of visual sensors with low energy resources or configured with low-quality codecs. On the other hand, such sensors should be more easily admitted to a region of interest with low relevance. Whatever the case, the sensors' positions are not relevant for the admission control, since it should be based on global-level priorities. As cameras with adjustable FoV may be deployed, potentially changing the priorities of source nodes, robust admission control mechanisms should be employed in such wireless visual sensor networks.

Global-level prioritization is primarily thought of as a way to differentiate visual source nodes according to their potential to provide relevant information for the applications. However, such a notion of prioritization may also be related to the confidentiality of some desired visual monitoring. In such a way, areas expected to have restricted monitoring access may be associated with higher priority levels, and an admission control mechanism may be employed to avoid unauthorized monitoring. Intermediate nodes may check some authorization key or flag to allow packet forwarding.

Wireless visual sensor networks may also face security attacks, such as denial of service (DoS). Sensor nodes have resource constraints that make them more vulnerable to such kinds of threat, especially when multimedia data is transmitted [36]. In fact, a DoS attack may be designed to compromise different aspects of the communications, for example increasing MAC layer collisions or providing malicious visual data to congest nodes. When exploiting global-level prioritization parameters, the network may try to disconnect parts of the network composed of lower relevant source nodes in order to preserve the most relevant sources. This principle may also be applied when implementing an intrusion detection system (IDS) [38]. In a different way, lower relevant source nodes may automatically cease transmission when a DoS is detected, reducing the amount of information that is being transmitted over the network, while higher relevant sources keep transmitting in order to not significantly impact the visual monitoring quality of the application.

## 6. Optimization Issues

We have discussed some promising investigation areas that can benefit from local and global-level prioritization parameters for improved performance. Nevertheless, some relevant issues should be considered when designing new optimization approaches following this trend.

In this section, we discuss three design issues that should be properly considered in new investigation works. Initially, we discuss the combination of optimization approaches, which may produce more efficient optimization solutions. After that, we argue that different optimizations may be employed to achieve equivalent results, and thus, it is worth analyzing the expected results and the application monitoring requirements. Finally, we believe that some global-level priority-based optimizations can be enhanced when local-level prioritization parameters are also exploited, potentially achieving better results.

### 6.1. Combining Optimizations

In general, global-level prioritization parameters may be exploited in many ways and during distinct stages of the network lifetime. Different results may be achieved depending on the design of the proposed optimization approach. Additionally, different optimizations could be combined to compose a more complete solution, potentially bringing more significant results.

As an example of this principle, both priority-based optimizations proposed in [7,22] could be combined to create a single solution. Actually, in [7], visual source nodes transmit images according to their sensing relevancies and the sub-bands resulting from a two-level 2D DWT. Considering that each image *i*, *i* = 0, …, *i*, sizes *B*_(_*_i_*_)_ bits, a mapping between (global-level) SR indexes [7] and the total amount of bits to transmit for every image *i* is described in Table 8.

A two-level 2D DWT is achieved processing the LL sub-band with a new 2D DWT, resulting in seven sub-bands: LL_(2)_, HL_(2)_, LH_(2)_, HH_(2)_, HL_(1)_, LH_(1)_, HH_(1)_. The LL_(2)_ sub-band is the most relevant for the reconstruction of the original images.

On the other hand, the work in [22] associates the SR indexes to the transmission frequency of each source node *s*, *s* = 0, …, *S*, which is referred to as *f*_(_*_s_*_)_. Adjusting the definitions of the sensing relevance in [22], which considered fewer values for SR, but the same groups of relevance, we achieve the definitions in Table 9.

As a novel approach, we could propose a combined operation of these optimizations, aiming at better performance related to energy consumption. The idea would be to exploit a global-level prioritization parameter to define the number and types of DWT sub-bands to be transmitted, as in [7], and the transmission frequency, as in [22]. The resulted optimizations may bring more significant results for wireless visual sensor networks.

That proposed priority-based enhanced monitoring is a valuable optimization for wireless visual sensor networks, but other optimizations could be proposed. Table 10 presents some ideas for novel combined operations of global-level priority-based optimizations.

### 6.2. Comparing Optimizations

Sometimes, an optimization goal may be achieved through different optimizations, and it is a relevant design issue choosing the most appropriate approach, which depends on the application monitoring requirements and the characteristics of the deployed network. In this context, it is worth comparing the expected results in order to evaluate what is the best approach for a particular monitoring application.

As an example, we could compare two different global-level priority-based optimizations for energy saving, which adapt different parts of the communication process. One of the optimizations proposed in [7] creates a direct mapping between the sensing relevance of source nodes and the total amount of information that source nodes have to transmit, according to DWT sub-bands, as described in Table 8. In fact, we can say that this optimization discards packets in advance, saving energy over the network. On the other hand, the optimization in [23] proposes a selective retransmission mechanism where some corrupted packets may not be retransmitted, according to the global-level priority of the transmitting sources. In other words, source nodes transmit the entire visual data (images), but some packets may not reach the sink, depending on the perceived packet error rate (PER). The proposed selective retransmission approach presented in [23] adds complexity to intermediate nodes, which must interpret the incoming packets when providing the appropriate transmission service. Additionally, if the PER is high, many packets transmitted in an unreliable way may not reach the sink, which may impact the visual quality of the reconstructed images. Table 11 presents an association between the SR indexes [7] and the expected transmission service.

The selective retransmission approach presented in [23] transmits more packets, and thus, it consumes more energy. However, this difference decreases for higher PER, since more corrupted low-relevant packets will not be retransmitted. Although the global-level priority-based transmission approach described in [7] seems to be most energy-efficient, fewer packets reach the sink on average when compared with the selective retransmission approach in [23]. Table 12 presents the expected average percentage of packet reception at the sink side, which places the selective retransmission as the best approach in terms of visual quality (although more effective mechanisms for quality measurement should still be considered). The PER_(h)_ refers to the average packet error rate at hop *h*, for a network composed of *H*_(_*_p_*_)_ + 1 hops [39].

There is then a tradeoff between energy consumption and the expected quality of reconstructed images. Thus, for this particular case, the application requirements should be considered when choosing the most appropriate approach in order to achieve higher performance.

Many other local or global-level priority-based approaches may have equivalent optimization goals, encouraging performance comparisons. Table 13 presents some example of such valuable comparisons.

### 6.3. Adding Local-Level Priority Parameters

A large set of optimizations may be proposed exploiting global-level prioritization parameters, but local-level prioritization approaches may also be exploited to complement the proposed optimization, potentially achieving higher performance.

Local-level relevancies can be defined based on different characteristics of the communications. In fact, such a type of relevance is significant only for the scope of the transmitting node, and thus, the way raw data is encoded may provide the required level of differentiation for prioritization mechanisms. A common approach is to give higher priority to parts of the encoded data that are more relevant for the reconstruction of the original data [9,40]. For image transmission, DWT coding may be exploited as presented in Figure 2, but other techniques, such as discrete cosine transform (DCT), are also promising. For video streaming, many codecs produce data with different relevancies, such as the H.264 codec. In fact, when exploiting H.264, I-frames may be assigned to higher relevance levels, since they are more critical for the reconstruction process. However, again, other techniques, such as distributed video coding (DVC) [9], may also be exploited. Whatever the case, local-level relevance may bring significant contributions to global-level priority-based optimizations. This subsection presents an example of how this information can be added to WVSN transmission approaches.

The work in [26] proposes a global-level priority-based packet-level redundancy mechanism aimed at improved error-resilience with low impact on the energy consumption. When avoiding retransmission of some corrupted packets, the work in [26] intends to reduce the average end-to-end transmission delay, potentially benefiting real-time monitoring applications. A simple replication-based transmission and a more complex transmission approach based on erasure coding are proposed in [26], and both of them only exploit the global-level sensing relevance concept, as defined in [7].

We define the number of replications of original data packets as *R*. If *R* = 1, every original data packet will be transmitted along with an additional copy, which will carry exactly the same payload. Table 14 presents the association between SR and *R* as defined in [26], for the replication-based transmission.

Considering that visual source nodes will transmit DWT-based images, we can propose an improved association between SR and *R*, as presented in Table 15.

For the most relevant source nodes (SR = 15), every original data packet will be transmitted along with two copies. For high-relevant sources (SR ranging from 11 to 14), one copy will be transmitted for every single data packet. The difference comes for the medium-relevant sources, where the replication transmission is valid only for the defined DWT sub-bands. The idea of this improved optimization is to increase the average quality of reconstructed images transmitted from medium-relevant source nodes, even though more energy is consumed.

Although more energy may be consumed over the network for the improved optimization, we can achieve higher quality for medium-relevant sources. Table 16 presents the average percentage of received packets for every transmitted image, concerning medium-relevant source nodes, where we can note that the combined optimization performs better. As commented on before, although it does not directly reflect the quality of reconstructed images, better quality may be achieved when more information is received considering the same DWT sub-band.

Local-level prioritization parameters may contribute to optimizations in wireless multimedia sensor networks, mainly when global-level relevancies are already exploited for prioritization. Table 17 presents some examples of global-level priority-based optimizations that can benefit from the adoption of local-level priority.

## 7. Conclusions

Different frameworks may be created to define local and global-level prioritization parameters, following characteristics, such as monitoring relevance, critical event detection, targets viewing, area coverage, multimedia coding, among others. In fact, such frameworks can be successfully defined in real-world wireless visual sensor networks, especially due to the nature of visual monitoring by camera-enabled sensors. Therefore, we can expect that real-world WVSN applications may be developed exploiting some prioritization parameters.

The stringent requirements of visual data transmission and the constrained nature of sensor networks will foster the development of optimizations that enhance the overall performance of the monitoring applications. Additionally, local or global-level prioritizations are central in such optimizations. In this context, we believe that this paper can significantly contribute to investigations where prioritization is a key design concept.

We have presented the main research trends when exploiting local and global-level prioritization, envisaging relevant research fields that could guide some investigation efforts in upcoming years. Actually, the discussed topics are intended to open promising research areas, which may bring valuable results to wireless visual sensor networks.

## Figures and Tables

**Table 1.** Some prioritization parameters.

**Parameter**	**Scope**	**Description**
Energy	Global	The energy level of the sensor node
Hardware capabilities	Global	Resources, such as the camera resolution, number and type of sensor units, memory capacity, *etc.*
Data type	Local or global	The transmitted data may be temperature, pressure, video stream, image snapshot, infrared image, audio stream, *etc.*
Media coding	Local	Codec and its configuration
Sensing relevance	Global	The relevance of the retrieved information for the monitoring functions of the application
Confidentiality	Local or global	Required confidentiality for the transmitted data
Criticality	Local or global	The level of criticality associated with the transmitted data

**Table 2.** Groups of relevance and the associated sensing relevance index (SR).

**Group of Relevance**	**SR**	**Description**
Irrelevant	0	The source node has no relevance for the application and should act only as a relay node.
Low relevance	1–4	Visual sensors are transmitting complementary visual information with low influence over the monitoring quality.
Medium relevance	5–10	The transmitted information is relevant, but some quality loss can be tolerated.
High relevance	11–14	Some visual sensors will have higher relevance for the application, requiring prioritized treatment by the network.
Maximum relevance	15	Monitoring quality is highly dependent on visual data transmitted by these source nodes.

**Table 3.** Load balancing for braided paths.

**Global-Level Priority**	**Maximum Number of Paths**
Very Low	0
Low	1
Average	2
High	3
Very High	4 or more

**Table 4.** Error recovery exploiting global-level prioritization.

**Global-Level Priority**	**Performed Optimization**
Very Low	No packet should be transmitted
Low	Unreliable transmission without correction codes
Average	Code rate of 0.9
High	Code rate of 0.7
Very High	Code rate of 0.5

**Table 5.** A hybrid optimized error recovery mechanism.

**Global-Level Priority**	**Performed Optimization**
0	No packet should be transmitted
1	Unreliable transmission without correction codes
2	Code rate of 0.9 for only the LL sub-band
3	Code rate of 0.9 for all packets
4	Code rate of 0.7 for only the LL sub-band
5	Code rate of 0.7 for all packets
6	Code rate of 0.6 for all packets
7	Code rate of 0.5 for all packets

**Table 6.** Adaptive reducing of the source transmission rate.

**Global-Level Priority**	**Source Behavior**
Low	Transmit only LL sub-band
Medium	Transmit only LL and HL sub-bands
High	Transmit all DWT sub-bands (full-quality images)

**Table 7.** Global-level priority-based secure transmission.

**Global-Level Priority**	**Source Behavior**
Low	Transmission of clear data
Medium	Periodic encryption with the DES (Data Encryption Standard) algorithm. Periods of encrypted data are intercalated with clear data transmissions
High	Continuous encryption with the DES algorithm
Very high	Continuous encryption with the 3DES algorithm

**Table 8.** Relevance-based energy-efficient image transmission.

**SR**	**Corresponding DWT Sub-Band(s)**	**Value of *B*_(_***_i_***_)_**
0	No packets to be transmitted.	0
1–4	LL_(2)_	*B*_(_*_i_*_)_/16
5–6	LL_(2)_ and HL_(2)_	*B*_(_*_i_*_)_/8
7–8	LL_(2)_, HL_(2)_ and LH_(2)_,	(*B*_(_*_i_*_)_*3)/16
9–10	LL_(2)_, HL_(2)_, LH_(2)_ and HH_(2)_	*B*_(_*_i_*_)_/4
11–12	LL_(2)_, HL_(2)_, LH_(2)_, HH_(2)_ and HL_(1)_	*B*_(_*_i_*_)_/2
13–14	LL_(2)_, HL_(2)_, LH_(2)_, HH_(2)_, HL_(1)_ and LH_(1)_	(*B*_(_*_i_*_)_*3)/4
15	LL_(2)_, HL_(2)_, LH_(2)_, HH_(2)_, HL_(1)_, LH_(1)_ and HH_(1)_	*B*_(_*_i_*_)_

**Table 9.** Relevance-based energy-efficient monitoring.

**SR**	***f*_(_***_s_***_)_**
0	0
1–4	0.1
5–6	0.3
7–8	0.4
9–10	0.5
11–12	0.6
13–14	0.8
15	1.0

**Table 10.** Some combined global-level priority-based optimizations.

**Optimization 1**	**Optimization 2**	**Combined Optimization**
Delay-aware routing [24]	Reliability based on packet-level redundancy [26]	More relevant visual sources will transmit packets through paths with lower end-to-end delay. Additionally, replicated packets are transmitted according to global-level priorities for improved reliability. The replicated packets could be transmitted through the same path or employing the remaining paths with lower expected delay.
Reliability based on packet-level redundancy [26]	Semi-reliable retransmission [23]	More relevant source nodes will transmit more replicated copies of data packets. Besides that, intermediate nodes will retransmit only corrupted packets from more relevant sources. Doing so, a high reliability level is achieved, but energy is saved due to unreliable transmissions from lower relevant visual sources.
Energy-efficient packet relaying [25]	Delay-aware routing [24]	Intermediate nodes will discard less relevant packets when their energy levels are below a pre-defined threshold. However, such behavior could be employed only in paths with lower end-to-end delay (best paths), enlarging their expected lifetime while reducing complexity over the network.

**Table 11.** Relevance-based selective retransmission.

**SR**	**DWT Sub-Band(s) Transmitted with Reliability Guarantees**
1–4	LL_(2)_
5–6	LL_(2)_ and HL_(2)_
7–8	LL_(2)_, HL_(2)_ and LH_(2)_
9–10	LL_(2)_, HL_(2)_, LH_(2)_ and HH_(2)_
11–12	LL_(2)_, HL_(2)_, LH_(2)_, HH_(2)_ and LH_(1)_
13–14	LL_(2)_, HL_(2)_, LH_(2)_, HH_(2)_, LH_(1)_ and HL_(1)_
15	LL_(2)_, HL_(2)_, LH_(2)_, HH_(2)_, LH_(1)_, HL_(1)_ and HH_(1)_

**Table 12.** Average percentage of received packets.

**SR**	**Global-Level Priority-Based Transmission [7] (%)**	**Global-Level Priority-Based Retransmission [23] (%)**
1–4	6.25	$6.25+93.75\times \left(\prod_{h=1}^{H_{\left(p\right)}+1}\left(1-PER_{\left(h\right)}\right)\right)$
5–6	12.5	$12.5+87.5\times \left(\prod_{h=1}^{H_{\left(p\right)}+1}\left(1-PER_{\left(h\right)}\right)\right)$
7–8	18.75	$18.75+81.25\times \left(\prod_{h=1}^{H_{\left(p\right)}+1}\left(1-PER_{\left(h\right)}\right)\right)$
9–10	25	$25+75\times \left(\prod_{h=1}^{H_{\left(p\right)}+1}\left(1-PER_{\left(h\right)}\right)\right)$
11–12	50	$50+50\times \left(\prod_{h=1}^{H_{\left(p\right)}+1}\left(1-PER_{\left(h\right)}\right)\right)$
13–14	75	$75+25\times \left(\prod_{h=1}^{H_{\left(p\right)}+1}\left(1-PER_{\left(h\right)}\right)\right)$
15	100	100

**Table 13.** Some valuable performance comparisons.

**Optimization 1**	**Optimization 2**	**Performance Comparisons**
Energy-efficient transmission [7]	Packet prioritization for congestion control [7]	When network faces congestion, some intermediate nodes may drop lower relevant packets. Packet discarding at source nodes or in congested nodes may bring different results in terms of energy consumption and monitoring quality, where the most appropriate approach depends on the perceived network congestion and the applications' monitoring requirements.
Reliability based on packet-level redundancy [26]	Priority-based retransmission [23]	Error recovery can be provided by different approaches, and sometimes, they may have equivalent results. Packet-level redundancy and hop-by-hop retransmission, when exploiting global-level prioritization parameters, may save energy while preserving the most relevant visual data for the applications.

**Table 14.** Packet-level redundancy transmission based on global-level priority.

**SR**	***R***
1–10	0
11–14	1
15	2

**Table 15.** SR- and DWT-based packet-level redundancy transmission.

**SR**	***R***	**Data that Will be Replicated**
1–4	0	-
5–6	1	LL sub-band
7–8	1	LL and HL sub-bands
9–10	1	LL, HL and LH sub-bands
11–14	1	Entire image
15	2	Entire image

**Table 16.** Average percentage of received packets for medium-relevant source nodes.

**SR**	**Redundancy Based on Global-Level Prioritization [26] (%)**	**Combined Optimization (%)**
5–6	$100\times \left(\prod_{h=1}^{H_{\left(p\right)}+1}\left(1-PER_{\left(h\right)}\right)\right)$	$25\times \left(\prod_{h=1}^{H_{\left(p\right)}+1}\left(1-\prod_{h=0}^{1}\left(PER_{\left(h\right)}\right)\right)\right)+75\times \left(\prod_{h=1}^{H_{\left(p\right)}+1}\left(1-PER_{\left(h\right)}\right)\right)$
7–8	$100\times \left(\prod_{h=1}^{H_{\left(p\right)}+1}\left(1-PER_{\left(h\right)}\right)\right)$	$50\times \left(\prod_{h=1}^{H_{\left(p\right)}+1}(1-\prod_{h=0}^{1}\left(PER_{\left(h\right)}\right))\right)+50\times \left(\prod_{h=1}^{H_{\left(p\right)}+1}\left(1-PER_{\left(h\right)}\right)\right)$
9–10	$100\times \left(\prod_{h=1}^{H_{\left(p\right)}+1}\left(1-PER_{\left(h\right)}\right)\right)$	$75\times \left(\prod_{h=1}^{H_{\left(p\right)}+1}(1-\prod_{h=0}^{1}\left(PER_{\left(h\right)}\right))\right)+25\times \left(\prod_{h=1}^{H_{\left(p\right)}+1}\left(1-PER_{\left(h\right)}\right)\right)$

**Table 17.** Some examples of combined optimizations based on local and global-level prioritization.

**Original Optimization**	**Optimization When Also Exploiting Local-Level Prioritization**
Delay-aware routing [24]	Local-level priorities can be exploited to enhance the routing policies of intermediate nodes. Doing so, global-level prioritization parameters and data coding relevancies would be considered when forwarding data packets.
Selective retransmission [23]	Packet retransmission could also consider local-level relevancies, achieving more complex levels of reliability. The work in [40] proposes a semi-reliable retransmission mechanism based only on DWT coding, which could be fundamental for the desired optimization.
